# Large Language Model Evaluation in Traditional Chinese Medicine for Stroke: Quantitative Benchmarking Study

**DOI:** 10.2196/81545

**Published:** 2025-12-11

**Authors:** Hulin Long, Yang Deng, Yaoguang Guo, Zifan Shen, Yuzhu Zhang, Ji Bao, Yang He

**Affiliations:** 1 Hospital of Chengdu University of Traditional Chinese Medicine Chengdu, Sichuan Province China; 2 Institute of Clinical Pathology West China Hospital of Sichuan University Chengdu China; 3 Physical Education Institute Southwest Medical University Luzhou China; 4 Chengdu University of Traditional Chinese Medicine Chengdu China

**Keywords:** large language models, traditional Chinese medicine, stroke, evaluation dataset, model evaluation, artificial intelligence

## Abstract

**Background:**

The application of large language models (LLMs) in medicine is rapidly advancing. However, evaluating LLM capabilities in specialized domains such as traditional Chinese medicine (TCM), which possesses a unique theoretical system and cognitive framework, remains a sizable challenge.

**Objective:**

This study aimed to provide an empirical evaluation of different LLM types in the specialized domain of TCM stroke.

**Methods:**

The Traditional Chinese Medicine-Stroke Evaluation Dataset (TCM-SED), a 203-question benchmark, was systematically constructed. The dataset includes 3 paradigms (short-answer questions, multiple-choice questions, and essay questions) and covers multiple knowledge dimensions, including diagnosis, pattern differentiation and treatment, herbal formulas, acupuncture, interpretation of classical texts, and patient communication. Gold standard answers were established through a multiexpert cross-validation and consensus process. The TCM-SED was subsequently used to comprehensively test 2 representative LLM models: GPT-4o (a leading international general-purpose model) and DeepSeek-R1 (a large model primarily trained on Chinese corpora).

**Results:**

The test results revealed a differentiation in model capabilities across cognitive levels. In objective sections emphasizing precise knowledge recall, DeepSeek-R1 comprehensively outperformed GPT-4o, achieving an accuracy lead of more than 17% in the multiple-choice section (96/137, 70.1% vs 72/137, 52.6%, respectively). Conversely, in the essay section, which tested knowledge integration and complex reasoning, GPT-4o’s performance notably surpassed that of DeepSeek-R1. For instance, in the *interpretation of classical texts* category, GPT-4o achieved a scoring rate of 90.5% (181/200), far exceeding DeepSeek-R1 (147/200, 73.5%).

**Conclusions:**

This empirical study demonstrates that Chinese-centric models have a substantial advantage in static knowledge tasks within the TCM domain, whereas leading general-purpose models exhibit stronger dynamic reasoning and content generation capabilities. The TCM-SED, developed as the benchmark for this study, serves as an effective quantitative tool for evaluating and selecting appropriate LLMs for TCM scenarios. It also offers a valuable data foundation and a new research direction for future model optimization and alignment.

## Introduction

In recent years, artificial intelligence technologies, particularly large language models (LLMs), have demonstrated immense potential in the health care sector [1]. LLMs have achieved substantial progress in applications, ranging from aiding clinical decision-making to optimizing patient communication [1]. The outstanding performance of general-purpose LLMs, such as ChatGPT (OpenAI), in natural language processing tasks suggests broad application prospects in complex medical scenarios [2]. However, traditional Chinese medicine (TCM) presents unique challenges for the application of LLMs [3,4]. TCM is a complex knowledge system with a unique theoretical framework, an emphasis on a holistic view (harmony between man and nature and pattern differentiation and treatment), and a reliance on highly individualized diagnosis and treatment plans [3,4].

To address these challenges, domain-specific TCM LLMs (eg, Huatuo and ShenNong) have been developed. Studies indicate that the performance of these specialized models on the Chinese National Medical Licensing Examination for TCM Practitioners is significantly superior to that of general-purpose models, highlighting the necessity of domain specialization [3]. This need for specialization, however, extends beyond technical data adaptation; at its core lie the profound epistemological differences between Chinese and Western medicine. The theoretical cornerstones of TCM, such as Yin-Yang and the Five Elements; Qi, Blood, and Body Fluids; and Zang-fu organs and meridians, constitute a unique cognitive framework with no direct correspondence to the ontology of modern medicine [4].

As an LLM’s understanding is fundamentally derived from statistical learning of text patterns, an effective evaluation dataset cannot be limited to recalling factual knowledge. Such a dataset must also be able to deeply assess a model’s ability to perform clinical reasoning within the specific framework of TCM. Therefore, the construction of this evaluation dataset is, in itself, a crucial step in translating the abstract TCM knowledge system into a modern, operational format. This process is vital for the digital representation, inheritance, and innovation of TCM knowledge.

General medical LLM evaluation datasets, such as those based on the United States Medical Licensing Examination [5], are insufficient for assessing a model’s depth and accuracy in understanding TCM-specific stroke concepts. Therefore, a specialized evaluation dataset for TCM stroke is of paramount importance. Such a dataset serves as both a necessary means to validate the accuracy and safety of models and a fundamental guarantee for guiding model iteration and building user trust.

Existing benchmark test sets often cover only a small fraction of a model’s potential output, thus limiting their ability to ensure the safety of health care LLMs [6]. It is crucial to evaluate LLMs in specific application scenarios; for TCM stroke, this requires testing them in specialized TCM contexts. The performance disparity between TCM LLMs and general LLMs on professional examinations [3] indicates that *domain adaptation* in highly specialized fields such as TCM is not a simple task. Merely fine-tuning with domain-specific texts may be insufficient [3], potentially necessitating architectural adjustments or innovative training methods to better capture the subtle, context-dependent knowledge of TCM. The abstract nature of TCM theory and its individualized features [4] may require LLMs to develop a deeper, more contextualized understanding than standard fine-tuning can provide. Consequently, a robust evaluation dataset can not only assess existing models but also drive research into more effective methods for imbuing LLMs with specialized, culturally rich medical knowledge.

The primary objective of this study is to conduct a rigorous empirical evaluation of leading LLMs within the specialized domain of TCM stroke. To enable this evaluation, we first undertook the systematic development of a high-quality, specialized benchmark, the Traditional Chinese Medicine-Stroke Evaluation Dataset (TCM-SED).

**Figure 1:**
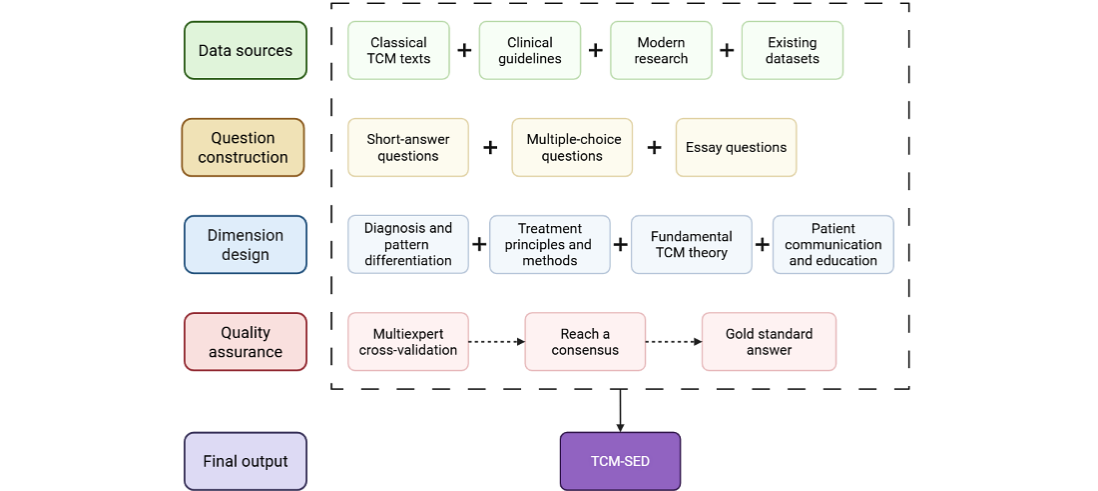
Flowchart for the construction of the Traditional Chinese Medicine-Stroke Evaluation Dataset. TCM: traditional Chinese medicine; TCM-SED: Traditional Chinese Medicine-Stroke Evaluation Dataset.

This paper describes the development methodology for this new dataset—covering data sourcing, question construction, and multiexpert validation (Figure 1)—and presents the results of our empirical study, which used the TCM-SED to compare the capabilities of GPT-4o (OpenAI) and DeepSeek-R1 (High-Flyer).

This research provides a direct, quantitative comparison of current LLM performance in TCM stroke. Furthermore, the resulting TCM-SED benchmark (and its described methodology) serves as a validated tool and foundation for future model development and evaluation in this specialized field.

## Methods

### Collection and Selection of Data Sources

The construction of a high-quality evaluation dataset required a multisource data system that was authoritative, classic, and cutting edge. The data sources for this study were derived from 2 levels, as described in the following paragraphs.

First, the Guidelines for the Diagnosis and Treatment of Cerebral Hemorrhage with Integrated Chinese and Western Medicine published by the Chinese Medical Association [7] were used as the core knowledge framework. These guidelines provided standardized diagnostic criteria, pattern differentiation, and treatment plans, serving as the cornerstone for ensuring the dataset’s content accuracy. Concurrently, to trace theoretical origins and ensure cultural depth, classical TCM texts (eg, Huangdi Neijing [Yellow Emperor’s Inner Canon] [8] and Jingui Yaolue [Synopsis of the Golden Chamber] [9]) as well as case records from renowned physicians were systematically reviewed. Pathological discussions, formula analyses, and treatment plans related to true stroke and stroke-like syndromes were extracted to ensure the dataset was deeply rooted in the theoretical foundations of TCM.

Second, modern scientific research findings and existing data resources were actively integrated. Modern research papers on the treatment of stroke with TCM were incorporated, with special attention paid to cutting-edge studies that correlate TCM patterns with biomedical indicators or imaging features (eg, computed tomography angiography and computed tomography perfusion) [10], thereby enhancing the scientific and contemporary nature of the dataset. Furthermore, existing public datasets were prudently reviewed. General medical question-answering datasets (eg, WebMedQA [11] and Huatuo-26M [12]) were examined to draw inspiration from their question structures. Specialized TCM datasets (eg, ShenNong-TCM-Dataset [13]) and question banks from the Chinese National Medical Licensing Examination for TCM Practitioners were analyzed to acquire professional question styles and content. TCM clinical case databases, if accessible and structurally processed, would also represent an invaluable supplementary data source [14].

To address the balance and sourcing of the 203 questions, our knowledge dimensions were systematically mapped to the primary data sources described in the previous two paragraphs. This process ensured that questions testing foundational theory were drawn from classical texts, while questions assessing clinical application were derived from modern guidelines and real-world cases. The primary data sources used for each knowledge dimension are summarized in Table 1.

**Table 1:** Primary data sources for the Traditional Chinese Medicine-Stroke Evaluation Dataset knowledge dimensions.

Knowledge dimension	Classical TCM^a^ books	Clinical guidelines	Existing question banks	Real clinical cases
Diagnosis and pattern differentiation		✓	✓	
Treatment principles and methods		✓	✓	
Fundamental TCM theory	✓			
Patient communication and education			✓	✓
Interpretation of classical texts	✓			
Clinical case analysis				✓

^a^TCM: traditional Chinese medicine.

### Evaluation Dimensions and Question Construction Paradigms

To comprehensively evaluate the capabilities of LLMs in the field of TCM stroke, 3 core question paradigms were designed: short-answer questions, multiple-choice questions, and essay questions.

Short-answer questions were primarily used to assess the model’s ability to master and recall core factual knowledge. These questions required the model to provide precise and concise answers (eg, “What are the main constituent herbs of Buyang Huanwu Tang?”). This paradigm served as a fundamental test of the model’s knowledge accuracy and has been applied in several TCM datasets, such as ShenNong [13].

Multiple-choice questions, including single-choice and multiple-choice formats, focused on evaluating the model’s ability to discriminate between similar or easily confused concepts. By designing distractors based on common misconceptions in TCM, the model’s precise grasp of knowledge points, such as diagnostic criteria and formula compatibility, could be effectively tested. This paradigm was widely adopted in mainstream evaluations such as MedQA [15] and ShenNong-TCM-EB [13] due to its objectivity and ease of automated scoring. As an example of this design principle, a question assessing the identification of TCM patterns in acute ischemic stroke was constructed to include a specific distractor based on a common misconception. While the correct options described valid acute patterns (eg, wind, phlegm, or blood stasis), the distractor was formulated as “Liver Qi stagnation is the most common pattern in the acute phase of stroke.” This distractor was specifically designed because liver Qi stagnation, while relevant to stroke, is typically associated with poststroke depression or chronic management, not the primary acute phase pattern. This design principle was applied throughout the dataset to test the model’s ability to accurately differentiate nuanced, context-dependent TCM concepts beyond simple recall.

Essay questions were designed to assess higher-order skills, such as knowledge integration, clinical reasoning, and text generation. These questions were typically based on a clinical case or a specific pathogenesis and required the model to conduct an in-depth analysis (eg, “Please discuss the pathogenesis, primary symptoms, and treatment principles and formulas for the pattern of liver-yang transforming into wind.”). By analyzing the long-form text answers generated by the model, its logical coherence, the rigor of its pattern-differentiation thinking, and its ability to apply knowledge could be examined, which were crucial for evaluating its potential as an assistant in real clinical scenarios [1,4].

In summary, by combining these 3 complementary question paradigms, a multilevel evaluation system was constructed to conduct a comprehensive and in-depth examination of LLMs across multiple dimensions, from knowledge recall and discrimination to integrated application.

### Design and Coverage of Evaluation Dimensions

To ensure the comprehensiveness of the evaluation, the question design of this dataset covered multiple key dimensions of knowledge in the field of TCM stroke. The core dimension was pattern differentiation and treatment (Bian-Zheng-Lun-Zhi), which required the model to perform pattern identification and differential diagnosis based on clinical manifestations, recommend formulas and make modifications, and select appropriate acupuncture points and explain their combination principles. On a theoretical level, the evaluation covered the understanding of pathological factors (eg, phlegm, blood stasis, wind, fire, and deficiency) and the ability to interpret relevant discussions in classical TCM texts, such as the Huangdi Neijing [8]. At the application and extension level, the dimensions were extended to rehabilitation, nursing care, and patient education. Among these, the evaluation of patient education and communication skills was considered particularly important, as the ability of an LLM to explain abstract TCM concepts in simple and accurate language was a key aspect of its value in real clinical settings [1].

### Expert Validation and Ground Truth Construction

The scientific validity and reliability of this evaluation dataset relied on the authoritativeness and uniqueness of its ground truth answers. To this end, a rigorous mechanism for expert validation and consensus building was established. The core of this process involved a review team composed of senior experts with extensive clinical experience in TCM stroke. To minimize individual bias and ensure the uniqueness of the final answers, the multireviewer cross-validation model advocated by the Real-World Evaluation of Large Language Models in Healthcare framework [5] was adopted.

This process was particularly critical for establishing the unique *gold standard answer* for all objective (short-answer and multiple-choice) questions. During the review, any initial disagreements on these answers were resolved through group discussion to reach a final consensus or were adopted directly from authoritative guidelines [1,7].

For the essay questions, which required subjective judgment, a separate process of independent expert scoring was used, as detailed in the Testing and Scoring Criteria section. To ensure consistency, detailed annotation guidelines and scoring rubrics were formulated in advance for the experts. Through this series of quality control measures, we ensured that the answer to every question in the dataset possessed the highest level of accuracy and authority.

### Testing and Scoring Criteria

The testing and scoring in this study adhered to the principles and standards outlined in the following paragraphs.

First, regarding the testing language, the entire evaluation dataset was presented in Chinese. Using Chinese was considered essential to ensure the highest degree of accuracy and professionalism, particularly for content involving pattern differentiation, formula rationale, and interpretation of classical texts, thereby assessing the model’s depth of understanding of core TCM concepts more effectively.

Second, regarding the test participants, this dataset was used to test 2 representative LLMs: GPT-4o and DeepSeek-R1. GPT-4o was chosen because, as one of the most advanced general-purpose large models internationally, it represents the current technological frontier. DeepSeek-R1 was chosen because, as a large model primarily trained on Chinese corpora, it has a unique advantage in handling tasks related to Chinese language and culture. Both models were the current mainstream versions of their respective series, and their test results hold broad reference value. All tests for both models were conducted within a controlled time frame (from June 16, 2025, to July 10, 2025) to ensure comparability of model versions.

Finally, regarding the scoring criteria, differentiated scoring methods were established for different question types to ensure objectivity and reliability:

- For short-answer and multiple-choice questions, a binary scoring method was used. A model’s answer was scored 1 point if it perfectly matched the unique gold standard answer, and 0 points otherwise.
- For essay questions, a rigorous dual-expert independent scoring method was used. Several key scoring points were predefined in the standard answer. Two senior TCM rehabilitation experts were then invited to independently assign a dynamic score (0-10 points) based on the model’s coverage and accuracy, without consulting each other.
- To quantitatively assess the consistency of this independent scoring, 2 measures of interrater reliability were calculated across all 40 responses. First, as a formal statistic accounting for chance agreement, Cohen weighted κ (with linear weights) was calculated due to the ordinal nature of the 0 to 10 scale. The analysis yielded κ value of 0.78, which indicates substantial agreement [16]. Second, as a descriptive measure of agreement, it was found that the exact agreement (ie, identical scores) was 38% (15/40), and the within 1-point agreement (ie, scores differed by 0 or 1 point) was 88% (35/40). Both metrics confirmed a robust and high degree of consistency in the independent scoring process.
- The final score for each category reported in the Essay Question Results section (with a maximum of 200 points) was calculated by summing the scores from both experts across all 10 questions in that category (10 questions×2 experts×10 points/question). This method allowed a comprehensive evaluation of the models’ integrated abilities by incorporating the independent judgments of both experts.

### Ethical Considerations

#### Institutional Review Board Approval and Exemption

This study was reviewed by the Scientific Research Department of the Hospital of Chengdu University of Traditional Chinese Medicine, which confirmed that the research complies with all medical ethics standards. The study was a secondary analysis and did not involve direct interaction with human participants or access to private patient-identifiable information. Thus, formal institutional review board approval was not required.

#### Informed Consent: Patients

The requirement for informed consent from patients was waived because the “real clinical cases” used in the clinical case analysis section were compiled from established medical textbooks, published case reports, and fully anonymized public datasets. The study involved no direct interaction with human participants.

#### Informed Consent and Compensation: Experts

The senior TCM experts who participated in the dataset validation and scoring process were provided with detailed information regarding the study's objectives and provided their informed consent to participate. Their participation was voluntary and uncompensated.

#### Privacy and Confidentiality

All data used in this study were fully deidentified and anonymized to ensure strict protection of privacy and confidentiality.

## Results

### Composition of the Evaluation Dataset

Through the systematic construction and validation process described in the Methods section, a specialized evaluation dataset for the field of TCM stroke was finalized and named the TCM-SED.

The TCM-SED comprised a total of 203 questions, structured into 3 core paradigms. The specific composition and purpose of each paradigm were as follows:

- Short-answer questions (46/203, 22.6%)—this section was designed to evaluate the accurate recall of core factual knowledge, covering diagnosis and pattern differentiation, treatment principles and methods, fundamental TCM theory, and patient communication.
- Multiple-choice questions (137/203, 67.4%)—this section, comprising both single- and multiple-choice formats, was focused on testing the model’s ability to discriminate between similar or easily confused concepts across the same key knowledge areas.
- Essay questions (20/203, 9.8%)—this section was used to assess higher-order analytic and reasoning skills. It was divided into 2 categories: interpretation of classical texts, which required models to analyze discussions from classical TCM literature, and clinical case analysis, which required models to conduct a full diagnostic and treatment process for a given scenario (including TCM diagnosis, treatment principle, prescription, and formula rationale).

This multilevel and multidimensional structure provided a solid data foundation for the subsequent comprehensive evaluation of LLM capabilities.

### Short-Answer Question Results

**Figure 2:**
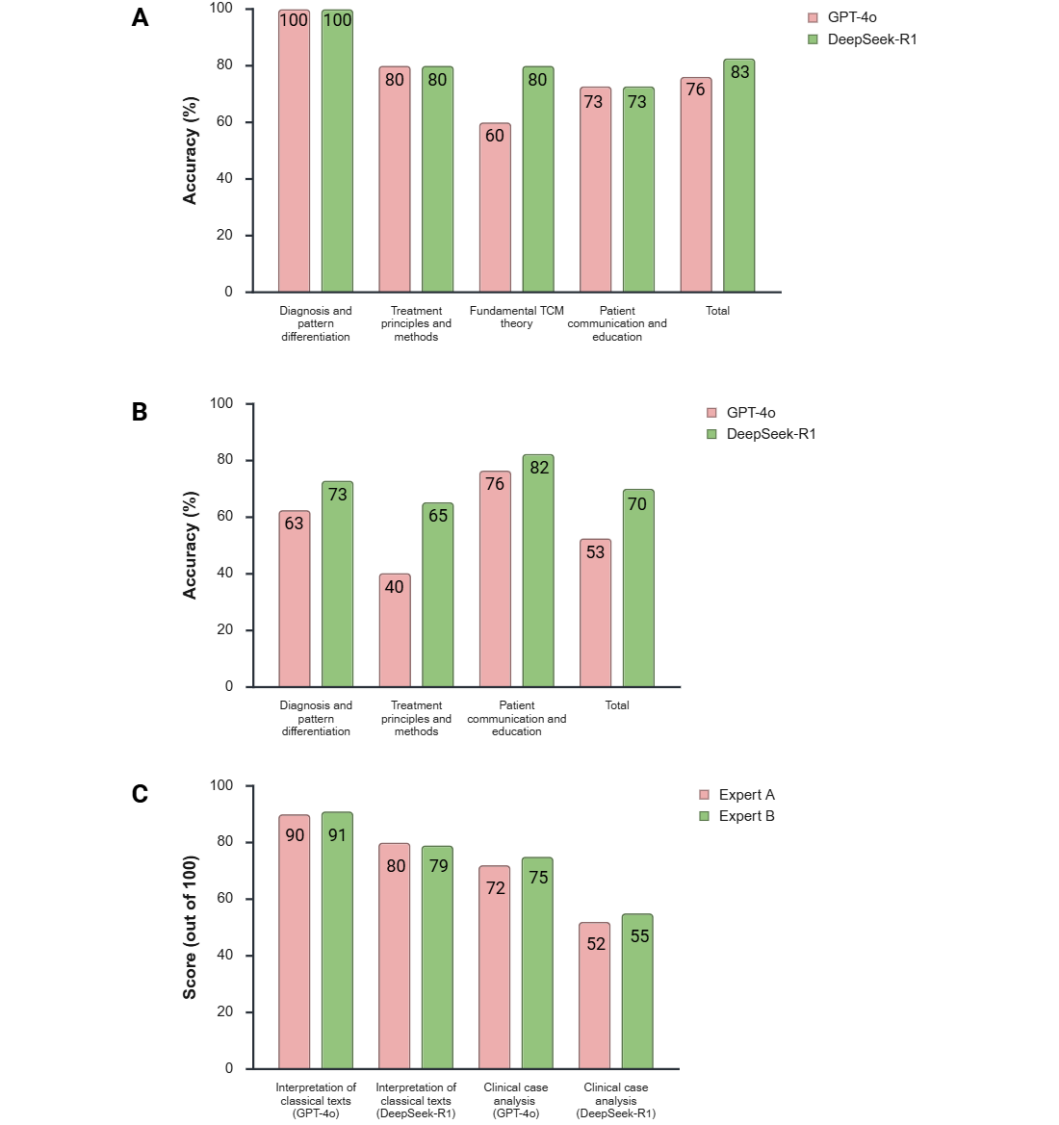
Performance comparison of GPT-4o and DeepSeek-R1 on the Traditional Chinese Medicine-Stroke Evaluation Dataset: (A) accuracy comparison on short-answer questions across different dimensions, (B) accuracy comparison on multiple-choice questions across different dimensions, and (C) grouped score comparison on essay questions, showing the separate total scores (out of 100) from each of the 2 independent expert raters to visualize interrater consistency. TCM: traditional Chinese medicine.

Overall, in the 46-question short-answer section, DeepSeek-R1 outperformed GPT-4o. DeepSeek-R1 achieved a total accuracy of 83% (38/46), while GPT-4o’s total accuracy was 76% (35/46). The performance of the 2 models varied across dimensions, as detailed in Figure 2A.

The test results indicated that in the diagnosis and pattern differentiation dimension, both models performed perfectly, achieving 100% (10/10) accuracy. In the treatment principles and methods and patient communication and education dimensions, the models’ performances were also identical, with accuracies of 80% (8/10) and 73% (8/11), respectively.

Notably, although the total scores in these latter categories were the same, the specific questions the models answered incorrectly were not identical, indicating distinct knowledge gaps. For instance, DeepSeek-R1 erred on a question regarding acupuncture point selection for poststroke speech impediment, which GPT-4o answered correctly. Conversely, GPT-4o erred on a question regarding the treatment method for a “phlegm-heat binding in the fu-organs” pattern, which DeepSeek-R1 answered correctly.

The most notable performance difference was observed in the *Fundamental TCM Theory* dimension. In this area, DeepSeek-R1’s accuracy was 80% (12/15), substantially higher than GPT’s 60% (9/15). This result suggested that while both models possessed similar capabilities in handling direct clinical application questions, the DeepSeek-R1 model demonstrated a stronger grasp of deeper, fundamental TCM theoretical knowledge.

### Multiple-Choice Question Results

In the 137-question multiple-choice section, the DeepSeek-R1 model’s performance was comprehensively superior to that of the GPT-4o model. DeepSeek-R1 achieved an overall accuracy of 70.1% (96/137), whereas GPT-4o’s overall accuracy was only 52.6% (72/137). The specific performance of the 2 models across different knowledge dimensions are detailed in Figure 2B.

The results showed that in the multiple-choice section, the performance gap between the 2 models widened further. DeepSeek-R1’s accuracy was approximately 17.5% higher than GPT-4o’s, indicating its clear advantage in handling TCM knowledge questions that required precise discrimination among distractors. The results from the multiple-choice questions further highlighted the advantage of domain-specific models (or those trained on a specific linguistic and cultural corpus) when dealing with highly specialized and nuanced knowledge.

Among the categories, the performance gap was most pronounced in the *Treatment Principles and Methods* dimension. DeepSeek-R1’s accuracy (47/72, 65%) was 25% higher than GPT-4o’s (29/72, 40%). This suggested that for clinical knowledge involving specific treatment plans and formula modifications that required detailed analysis, the DeepSeek-R1 model, trained on a core of Chinese corpora, possessed a deeper and more accurate understanding.

The performance of both models in the *patient communication and education* dimension was the highest among their respective categories, reaching 76% (13/17) for GPT-4o and 82% (14/17) for DeepSeek. This reflected that general-purpose large models possessed a relatively good foundational ability in language organization and layperson explanation.

### Essay Question Results

The essay question section, consisting of 20 questions (10 for *interpretation of classical texts* and 10 for *clinical case analysis*), was designed to assess higher-order skills. As described in the Testing and Scoring Criteria section, 2 senior TCM experts independently scored each question (from 0 to 10 points). The total score for each category (maximum 200 points) represented the sum of the independent scores from both experts across all 10 questions. This scoring method, which demonstrated *substantial* interrater reliability (κ=0.78), was used to generate the final results.

The test results showed a trend completely opposite to that of the short-answer and multiple-choice questions, as GPT-4o’s performance was comprehensively superior to that of DeepSeek-R1’s performance. Detailed visual comparison of the scores from each independent expert is shown in Figure 2C.

In the assessment of comprehensive exposition skills, GPT-4o performed markedly better than DeepSeek-R1. In the *interpretation of classical texts* category, GPT-4o achieved a scoring rate of 90.5% (181/200), far exceeding DeepSeek-R1’s scoring rate of 73.5% (147/200). In the more complex *clinical case analysis*, GPT-4o’s scoring rate (159/200, 79.5%) was also substantially ahead of DeepSeek-R1’s scoring rate (107/200, 53.5%).

This result highlighted the differences in the core capabilities of the 2 models. Although DeepSeek-R1 had an advantage in the precise recall of factual knowledge, especially in multiple-choice questions, GPT-4o demonstrated stronger abilities in long-text generation, logical organization, knowledge integration, and complex reasoning. Essay questions required not only accurate knowledge points but also the ability of the model to effectively organize these points into a coherent and rigorous analysis. As a leading international general-purpose large model, GPT-4o’s powerful underlying generation and reasoning capabilities enabled it to perform better on such tasks.

## Discussion

This study presented an empirical evaluation of 2 representative LLMs, GPT-4o and DeepSeek-R1, within the specialized domain of TCM stroke. The evaluation, which was conducted using the newly developed TCM-SED benchmark, revealed notable differences in their performance capabilities and limitations within this professional domain.

### Core Findings and Interpretation

The most central finding of this evaluation was that the DeepSeek-R1 model, primarily trained on Chinese corpora, demonstrated a comprehensively and substantially superior performance compared to the internationally leading general-purpose large model, GPT-4o. This advantage was particularly prominent in the multiple-choice section, which required precise discrimination, with a total accuracy gap of more than 17%.

This difference was attributed primarily to the underlying disparities in their training data. TCM is a knowledge system deeply intertwined with Chinese language, culture, and philosophical thought. The training of the DeepSeek-R1 model on massive Chinese corpora provided it with a more *native* understanding of TCM terminology, context, and unique logical paradigms (eg, pattern-differentiation thinking). In contrast, the general-purpose model GPT-4o appeared to rely more on a *translational* understanding when handling these issues. This *translational* approach, while capable of managing direct, factual short-answer questions, proved insufficient when required to distinguish subtleties in multiple-choice questions, especially those with carefully designed distractors based on TCM theory.

This analogy was used to describe the models’ differing proficiency in statistical pattern matching, rather than implying genuine cognitive comprehension. This distinction is critical because core TCM concepts, such as “Yin-Yang and Five Elements” and “Qi,” lack direct, one-to-one equivalents in English. A model relying on a *translational* framework might fail to grasp the nuanced, context-dependent application of these foundational concepts. In contrast, a *native* model, trained extensively on the source Chinese corpus, could more accurately replicate the correct contextual patterns and theoretical linkages, even without genuine cognitive comprehension.

The performance differences across evaluation dimensions further corroborated this judgment. In the short-answer questions on *diagnosis and pattern differentiation*, both models performed perfectly, indicating that both possessed strong memory and recall abilities for clear, structured knowledge from guidelines. However, when it came to the deeper *Fundamental TCM Theory* and the more nuanced *Treatment Principles and Methods*, especially in the multiple-choice questions, the superiority of DeepSeek-R1 became evident. This suggested a capability gap between *knowing what* (ie, the diagnosis) and *knowing why* (ie, understanding the underlying theory and principles), a gap identified as crucial for evaluating TCM LLMs.

However, the results of the essay questions showed a completely opposite trend, revealing a deep-seated differentiation in the capabilities of the 2 models. In both *interpretation of classical texts* and *clinical case analysis* tasks, GPT-4o’s performed substantially better than DeepSeek-R1. This finding was interpreted to highlight the core capability differences between the 2 models. Short-answer and multiple-choice questions focused more on the precise recall and discrimination of *static knowledge*, where DeepSeek-R1 excelled due to its *native* advantage in Chinese corpora. In contrast, essay questions required a strong *dynamic capability* to logically organize and integrate disparate knowledge points and to generate well-structured, coherent long-form text. As a leading general-purpose large model, GPT-4o’s powerful underlying reasoning and text generation capabilities enabled it to perform better on such complex, open-ended analytical tasks. This finding provided important insights into the applicable scenarios for different models: one model was more akin to a knowledgeable *database*, while the other was more like an adept *reasoning engine*.

### Importance and Application Value

The importance of this study is 2-fold.

First, for model development, the results provided clear evidence regarding the development of large models in highly specialized and culturally rich fields such TCM. This evidence suggests that merely fine-tuning a general-purpose model may be insufficient and that the foundational pretraining data are of critical importance. Future research and development should focus more on constructing high-quality, Chinese pretraining corpora that contain deep domain knowledge.

Second, for model evaluation, the TCM-SED, which was constructed for this study, proved to be both effective and necessary. It successfully identified subtle differences in the professional capabilities of different models, providing a quantifiable tool for assessing the safety and reliability of TCM LLMs. This dataset can serve as a benchmark for future iterations of related models, helping developers identify their models’ shortcomings.

### Limitations and Future Outlook

This study also had certain limitations. First, while the dataset’s size (N=203) was sufficient to reveal trends, it was relatively limited. This scale was determined as a pragmatic balance between achieving comprehensive coverage of all core knowledge dimensions (as shown in Table 1) and the substantial time and resource investment required for the rigorous multiexpert validation process. A larger-scale dataset would provide more statistically significant results. Second, this study focused solely on the disease of stroke, and its conclusions require further validation to determine their generalizability to other areas of TCM (eg, internal medicine, gynecology, or pediatrics). Finally, regarding the scoring of essay questions, while our dual-expert method demonstrated *substantial* reliability (κ=0.78), a degree of subjectivity in human annotation cannot be entirely eliminated. This limitation directly informs our proposed future work on exploring methods that combine LLM-assisted evaluation with expert assessment to further improve scoring consistency.

Looking ahead, several directions are proposed:

- Dataset expansion—the question bank can be further expanded in both depth and breadth, incorporating more diseases and introducing more diverse question types, such as multimodal diagnosis.
- Optimization of scoring mechanisms—methods that combine LLM-assisted evaluation with expert assessment should be explored to improve the efficiency and consistency of scoring open-ended questions.
- Dynamic and adversarial evaluation—more challenging dynamic evaluation benchmarks could be developed, such as simulating continuous, multiturn physician-patient dialogues or introducing adversarial questions designed to attack a model’s knowledge gaps, to more comprehensively assess model robustness and safety.
- Empowering model optimization and alignment—the TCM-SED is not only a measuring stick but can also serve as a high-quality training set to provide feedback for model improvement. Its expert-validated question-answer pairs can be directly used for supervised fine-tuning to correct model errors on specific knowledge points. In particular, the expert scoring results from the essay questions provide valuable data for building reward models and conducting reinforcement learning from human feedback, which can help achieve a deeper alignment between the model’s output and the clinical thinking patterns and value judgments of TCM experts, thereby genuinely enhancing their professional capabilities.

### Conclusions

This empirical study rigorously evaluated leading LLMs (GPT-4o and DeepSeek-R1) in the specialized domain of TCM stroke, using the newly developed and expert-validated TCM-SED benchmark.

The findings revealed a clear differentiation in capabilities: the Chinese-centric model (DeepSeek-R1) demonstrated superior performance in *static knowledge* tasks requiring precise recall, whereas the general-purpose model (GPT-4o) excelled in *dynamic reasoning* and complex generation tasks. This highlights the critical importance of culturally and linguistically specific pretraining data for specialized medical domains. The TCM-SED, validated through this process, is now established as an effective tool for future benchmarking, selection, and alignment of LLMs in TCM.

## Abbreviations

LLM: large language model
TCM: traditional Chinese medicine
TCM-SED: Traditional Chinese Medicine-Stroke Evaluation Dataset

## Data Availability

The datasets generated or analyzed during this study are available in the GitHub repository [17].
